# Associations of the gut mycobiome and its cross-kingdom interactions with estrus return in post-weaning sows revealed by metagenomic analysis

**DOI:** 10.3389/fmicb.2026.1892244

**Published:** 2026-09-03

**Authors:** Pan Jiang, Mengqing Zhou, Yuhong Liao, Wenbo Du, Min Liu

**Affiliations:** National Key Laboratory of Swine Genetic Improvement and Germplasm Innovation, Jiangxi Agricultural University, Nanchang, China

**Keywords:** cross-kingdom interactions, estrus return, gut mycobiome, metagenomics, multi-omics analysis, post-weaning sow

## Abstract

Post-weaning estrus return is a critical determinant of reproductive efficiency in the swine industry. While the gut microbiome, particularly bacteria, has been significantly associated with estrus return in sows, the role of the gut mycobiome and its cross-kingdom interactions with bacteria in this context remains largely unexplored. Here, we employed fecal metagenomics to characterize the gut mycobiome in 85 sows and investigated its association with post-weaning estrus return. A total of 22 fungal species were significantly associated with estrus return. Normal-return sows were characterized by increased abundance of *Arxiozyma slooffiae* (formerly *Kazachstania slooffiae*) and decreased abundances of *Malassezia pachydermatis* and *Alternaria rosae*. Moreover, we uncovered cross-kingdom interactions between fungi and bacteria associated with estrus return, where *Arxiozyma slooffiae* showed a positive correlation with *Prevotella* spp. enriched in normal-return sows. These interactions were predicted to involve the exchange of metabolites, including Fe^2+^, thiamine, and nicotinate. Fungal biomarkers demonstrated good discriminatory power for distinguishing normal-return and non-return sows (AUC = 0.906), and the combination with bacterial biomarkers further enhanced the performance (AUC = 0.947). Integrated multi-omics analysis revealed extensive associations between gut fungi and hormones and hormone-related compounds, as well as microbial functional pathways. Notably, *Arxiozyma slooffiae* was positively correlated with phytoestrogens (including daidzein and genistein) and the steroid hormone biosynthesis pathway but negatively correlated with testosterone. Collectively, these findings provide comprehensive insights into the role of the gut mycobiome and its cross-kingdom interactions in sow reproductive performance.

## 1 Introduction

The weaning-to-estrus interval (WEI) is a key indicator of reproductive efficiency in swine production. Although genetic selection and optimized management have significantly reduced the average WEI from 11.5–20.5 days in the 1980s to the current 5–7 days (Bryant et al., 1985; Johnston et al., 1986), approximately 15% of sows fail to return to estrus within 7 days post-weaning (Poleze et al., 2006). This delayed return to estrus increases non-productive days (NPD) and significantly reduces the number of piglets per sow per year (PSY), causing significant economic losses in the swine industry. Post-weaning return to estrus in sows is primarily driven by hormonal changes triggered by the cessation of lactation, but the timing and success of this process are frequently influenced by multiple factors, including nutrition, genetics, and management conditions (Almond, 1992). Accumulating evidence indicates that the gut microbiota plays a crucial role in regulating host reproductive function (He and Li, 2020; Munyoki et al., 2025). Notably, our previous study showed that the gut bacteria *Prevotella* spp. influence post-weaning sow return to estrus by modulating the metabolism of sex steroid hormones (Liu et al., 2023). It has been reported that supplementation with *Bacillus* and isomalto-oligosaccharides reshapes the gut microbiota composition and significantly shortens the WEI (Gu et al., 2021). Collectively, these findings highlight the pivotal role of the gut microbiota in sow reproduction.

The gut microbiota is a complex ecological community that harbors diverse microorganisms. Although fungi account for less than 1% of the gut microbial community (Arumugam et al., 2011; Nash et al., 2017; Huang et al., 2024), accumulating evidence indicates that they not only participate in the regulation of host physiological processes but also play critical roles in shaping intestinal immune responses (Doron et al., 2021; Li et al., 2022; Yan et al., 2024). Notably, it has been reported that *Aspergillus tubingensis* can promote the development of polycystic ovary syndrome through its metabolic products (Wu et al., 2025), suggesting that gut fungi may be directly involved in reproductive function. The fungal community in the pig gut is primarily composed of the phyla Ascomycota and Basidiomycota, with certain yeast species persisting stably and serving as core members that maintain intestinal homeostasis (Ramayo-Caldas et al., 2020). Researchers have demonstrated that supplementation of pig diets with *Saccharomyces cerevisiae* improves production performance and promotes health by suppressing pathogenic bacteria (Elghandour et al., 2020). Furthermore, the core fungal species *Kazachstania slooffiae* has been reported to promote intestinal epithelial glycolysis via SIRT5-dependent lysine desuccinylation, revealing a mechanism by which gut fungi regulate host metabolism (Hu et al., 2023). Following a recent taxonomic revision, this species has been formally transferred to the genus *Arxiozyma* and is now recognized as *Arxiozyma slooffiae* (Liu et al., 2024). Beyond these beneficial roles, specific fungal taxa have been associated with post-weaning diarrhea (Ren et al., 2025). These findings highlight the critical importance of gut fungi in regulating physiological metabolism and maintaining health in pigs.

Meanwhile, fungi serve as keystone species in maintaining the structural and metabolic stability of the gut microbiome, exerting ecological impacts that far exceed their proportion within the microbial community (Kumamoto, 2016). Gut fungi and bacteria can engage in complex cross-kingdom interactions through direct mechanisms (cellular contact and metabolite crosstalk) and indirect mechanisms mediated by host immunity (Zhang et al., 2022). Studies have shown that fungal-bacterial interactions are significantly enhanced in the intestines of colorectal cancer patients and correlate positively with disease progression (Liu et al., 2022), suggesting the critical role of gut bacteria–fungi cross-kingdom interactions in host physiology and health. Notably, the gut mycobiome of weaned piglets and its interactions with bacterial communities have been characterized, implying that cross-kingdom interactions may significantly impact long-term growth and health (Arfken et al., 2019; Lin et al., 2022). However, whether gut fungi and their interactions with bacteria influence sow reproductive performance remains largely unexplored.

Over the past decades, 18S rRNA gene and internal transcribed spacer (ITS) region sequencing have been the conventional approaches for investigating gut mycobiome composition. Nevertheless, the recent expansion of fungal reference genomes and methodological innovation have enabled metagenomic sequencing to emerge as an alternative approach for comprehensive mycobiome investigation (Lind and Pollard, 2021). In humans, a large-scale metagenomic analysis of over 10,000 fecal samples systematically characterized the structural features of gut mycobiome profiles across populations and identified disease-associated fungal biomarkers (Yan et al., 2024). Additionally, a study involving 816 fecal metagenomes systematically mapped the gut fungal landscape in colorectal cancer and uncovered complex cross-kingdom interactions (Yinhang et al., 2026). These advances support the application of metagenomic approaches to investigate gut fungal diversity and its association with sow reproductive performance.

In this study, we employed metagenomic sequencing to characterize the gut mycobiome composition in sows and to identify key fungal signatures associated with post-weaning estrus. We further analyzed fungal-bacterial interactions and evaluated the potential of a combined fungal and bacterial biomarker panel for discriminating between non-return and normal-return sows. Moreover, we aimed to delineate the relationships among the gut mycobiome, gut microbial functionality, fecal metabolome, and post-weaning estrus, thereby providing comprehensive insights into the role of the gut mycobiota and its cross-kingdom interactions in sow reproductive performance.

## 2 Materials and methods

### 2.1 Animals and sample collection

A total of 114 Landrace × Large White sows with an average parity of overall 5.54 ± 1.85 were obtained from two independent commercial farms located in Fujian Province, China. These two farms provided the discovery cohort of 85 sows and the independent validation cohort of 29 sows, respectively. Specifically, parity was well-balanced between the normal group (5.24 ± 1.79) and the non-return group (4.84 ± 2.1), with no significant difference observed (*p* > 0.05, Wilcoxon rank-sum test; Supplementary Table S3). All sows were clinically healthy with no signs of disease and had exhibited normal reproductive performance in previous cycles, including regular estrus and normal farrowing. The sows were managed under standardized commercial production systems, transitioning from individual farrowing crates during lactation to group-housed gestation pens after weaning. All sows were weaned at exactly 28 days of lactation. Both farms utilized enclosed barns with mechanical ventilation systems, and sows had *ad libitum* access to water via nipple drinkers. The sows were fed identical commercial diets (67% corn, 26% soybean meal, 1% soybean oil, and 6% premix), with the nutrient composition detailed in Supplementary Table S1. Prior to sample collection, the sows had not received antibiotics or probiotics for at least 2 months. Fecal samples were obtained in the afternoon on the day of weaning and immediately snap-frozen in liquid nitrogen before being stored at −80 °C for DNA extraction and metabolomic analyses. Following sample collection, estrus detection was performed twice daily (8.00 a.m. and 4.00 p.m.) by assessing vulvar swelling, redness, and the standing reflex in response to back pressure. Based on the recorded WEI, sows were categorized into the normal group (return to estrus within 7 days post-weaning; discovery cohort: *n* = 45; validation cohort: *n* = 18) and the non-return group (failed to return to estrus within 14 days post-weaning; discovery cohort: *n* = 40; validation cohort: *n* = 11). Sows returning to estrus between days 8 and 14 post-weaning were excluded from the analysis.

### 2.2 DNA extraction and shotgun metagenomic sequencing

Microbial DNA extraction and quality evaluation were conducted according to previously published protocols (Liu et al., 2023). Briefly, total DNA was isolated from fecal samples using the E. Z. N. A.® Soil DNA Kit (Omega Bio-Tek, Norcross, GA, USA). DNA integrity and concentration were examined by agarose gel electrophoresis and NanoDrop® spectrophotometry, respectively. For library preparation, genomic DNA was sheared to an average fragment size of approximately 400 bp using a Covaris M220 system (Gene Company Ltd., Hong Kong, China). Paired-end sequencing libraries were prepared with the NEXTFLEX Rapid DNA-Seq Kit (Bioo Scientific, Austin, TX, USA) and sequenced on an Illumina NovaSeq 6,000 platform (Illumina, San Diego, CA, USA) at Majorbio Bio-Pharm Technology Co., Ltd. (Shanghai, China), generating 150 bp paired-end reads according to manufacturer’s instructions.

### 2.3 Quality control and preprocessing of metagenomic sequences

Raw sequencing reads were processed to remove adapters and low-quality bases using fastp (v0.20.0) (Chen et al., 2018). Host contamination was subsequently eliminated by aligning reads against the swine reference genome (Sscrofa11.1) using BWA-MEM2 (v2.2.1) (Vasimuddin et al., 2019), and clean reads were retained for downstream analysis.

### 2.4 Fungal community analysis

For taxonomic classification, a custom fungal reference database was constructed. Briefly, all available fungal genomes were retrieved from the NCBI RefSeq database (accessed on December 11, 2025). In addition, the reference genome of *Arxiozyma slooffiae* (GenBank assembly GCA_017347545.2), a well-documented fungal taxon in the pig gut, was manually incorporated. The custom taxonomic index was subsequently generated using the kraken2-build utility from Kraken2 (v2.1.6) (Wood et al., 2019). In total, 666 curated fungal genomes were included in the library for taxonomic assignment. Clean reads were analyzed using Kraken2 (v2.1.6) with default parameters, and the relative abundance of fungal species was estimated using Bracken (v3.0.1) (Lu et al., 2017) with a read length of 150 bp. Altogether, 662 fungal species were detected, of which those with an average relative abundance > 0.1% and a prevalence > 20% across all samples were retained, yielding 652 fungal species for downstream analyses. GeneMark-ES (v4.7.1) (Lomsadze et al., 2005) was used to perform gene prediction on fungal genomes.

### 2.5 Bacterial community analysis

Single-sample binning was performed using MetaBAT 2 (v2.12.1, parameters: -m 1,500) (Kang et al., 2019) and VAMB (v3.0.2, parameters: -m 1,500 --minfasta 200,000) (Nissen et al., 2021). The resulting bins were analyzed using CheckM (v1.1.3) (Parks et al., 2015) to evaluate genome completeness and contamination, with putative bins showing ≥ 50% completeness and ≤ 5% contamination retained. These metagenome-assembled genomes (MAGs) were subsequently dereplicated using dRep (v2.6.2, parameters: -comp 50 -con 5 -nc 0.25 -pa 0.9 -sa 0.99) (Olm et al., 2017), yielding 2,704 non-redundant genomes. Taxonomic annotation of the non-redundant MAGs was performed using GTDB-Tk (v2.4.0) (Chaumeil et al., 2020) based on the GTDB database release 220. The abundance of each MAG across samples was then quantified using the quant_bins module integrated with Salmon in metaWRAP (v1.2.1) (Uritskiy et al., 2018). MAGs with an average relative abundance < 0.1% and a prevalence < 20% across all samples were excluded, resulting in a final set of 1,523 bacterial MAGs. Of these, high-quality MAGs (HQMAGs, completeness > 90% and contamination < 5%) were used for differential abundance analysis between groups.

### 2.6 Cross-kingdom associations analysis between fungi and bacteria

Spearman correlation analysis was performed to evaluate the associations between bacterial and fungal richness. An inter-kingdom correlation network was constructed based on the abundances of estrus-associated bacterial and fungal taxa. Significant associations were identified using Spearman correlation coefficients, with the Benjamini-Hochberg (BH) correction applied to control the false discovery rate (FDR). Only correlations with an absolute Spearman correlation coefficient (∣*rho*∣) greater than or equal to 0.4 and an FDR less than 0.05 were retained. The resulting inter-kingdom network was visualized using Cytoscape (v3.10.3) (Shannon et al., 2003).

### 2.7 Building genome-scale metabolic models and identifying metabolic interactions among fungi and bacteria

Genome-scale metabolic models (GEMs) were reconstructed for high-quality bacterial MAGs and fungal genomes. Predicted protein-coding sequences were used as input for model reconstruction with CarveMe (v1.6.2) (Machado et al., 2018) using default parameters. Pairwise cross-kingdom metabolic interactions were evaluated using SMETANA (v1.2.0) (Zelezniak et al., 2015) in global mode. Metabolic interaction potential (MIP) and metabolic resource overlap (MRO) were calculated to quantify cooperative metabolic exchange and resource competition, respectively. To further characterize specific metabolic interactions, SMETANA was applied in detailed mode to examine fungal-bacterial metabolic exchanges. Metabolite uptake scores (MUS) were calculated to quantify the growth dependency of each organism on specific metabolites. Metabolites with MUS > 0.9 were considered essential for the survival of fungi and bacteria.

### 2.8 Data statistical analysis

#### 2.8.1 Diversity and differential abundance analyses

Fungal rarefaction curves, alpha diversity indices (Shannon and Simpson), and beta diversity based on Bray-Curtis distances were calculated using the vegan (v2.7.1) R package. Group differences in alpha diversity were assessed using the Wilcoxon rank-sum test, while beta diversity was analyzed using PERMANOVA. To evaluate whether sow parity influenced the gut fungal community, this variable was included in the PERMANOVA analysis. Differentially abundant fungal species and high-quality bacterial MAGs between the groups were identified using linear discriminant analysis effect size (LEfSe) (Segata et al., 2011) analysis, with an LDA score ≥ 2 and an FDR-adjusted *p* < 0.05. Furthermore, multivariate logistic regression was performed to validate the associations between differential fungal taxa and estrus return while adjusting for parity.

#### 2.8.2 Random forest classification model

Random Forest (RF) models were constructed using the randomForest (v4.7.1.2) (Breiman, 2001) R package, with model performance evaluated via the caret (v7.0.1) and pROC (v1.18.5) (Robin et al., 2011) packages. Within the discovery cohort, optimal hyperparameters (mtry and ntree) were selected by grid search strategy to minimize the out-of-bag (OOB) error. The internal stability and performance of models were then assessed using leave-one-out cross-validation (LOOCV). Based on these optimized parameters, a final RF model was finalized using the complete discovery cohort and directly applied to the independent validation cohort without any further modification. To ensure consistency between datasets, validation samples were aligned to the feature space of the discovery cohort, with absent taxa assigned an abundance value of zero. Model performance across both cohorts was comprehensively assessed using AUC, accuracy, sensitivity, specificity, positive predictive value (PPV), and negative predictive value (NPV). The same pipeline was applied to the combined fungi-bacteria model to evaluate its predictive potential.

#### 2.8.3 Association analysis among fungal species, functional potential, and metabolites

The abundances of fungal species, KEGG pathways, and metabolites were log_10_ (x + 1) transformed and Z-score standardized. The KEGG pathways and fecal hormone and hormone-related metabolites significantly associated with the return to estrus were derived from our previous study, in which the detailed methodologies for their identification and quantification were described (Liu et al., 2023). These data were generated from the same fecal samples collected from the discovery cohort (*n* = 85) at the time of weaning. Procrustes analysis and Spearman correlation analysis were performed to assess the associations among fungal species, metabolites, and KEGG pathways. *p*-values were adjusted using the Benjamini-Hochberg FDR method. All data visualizations were generated using the ggplot2 (v3.5.2) package.

## 3 Results

### 3.1 Overview of the gut mycobiome composition in post-weaning sows

**Figure 1:**
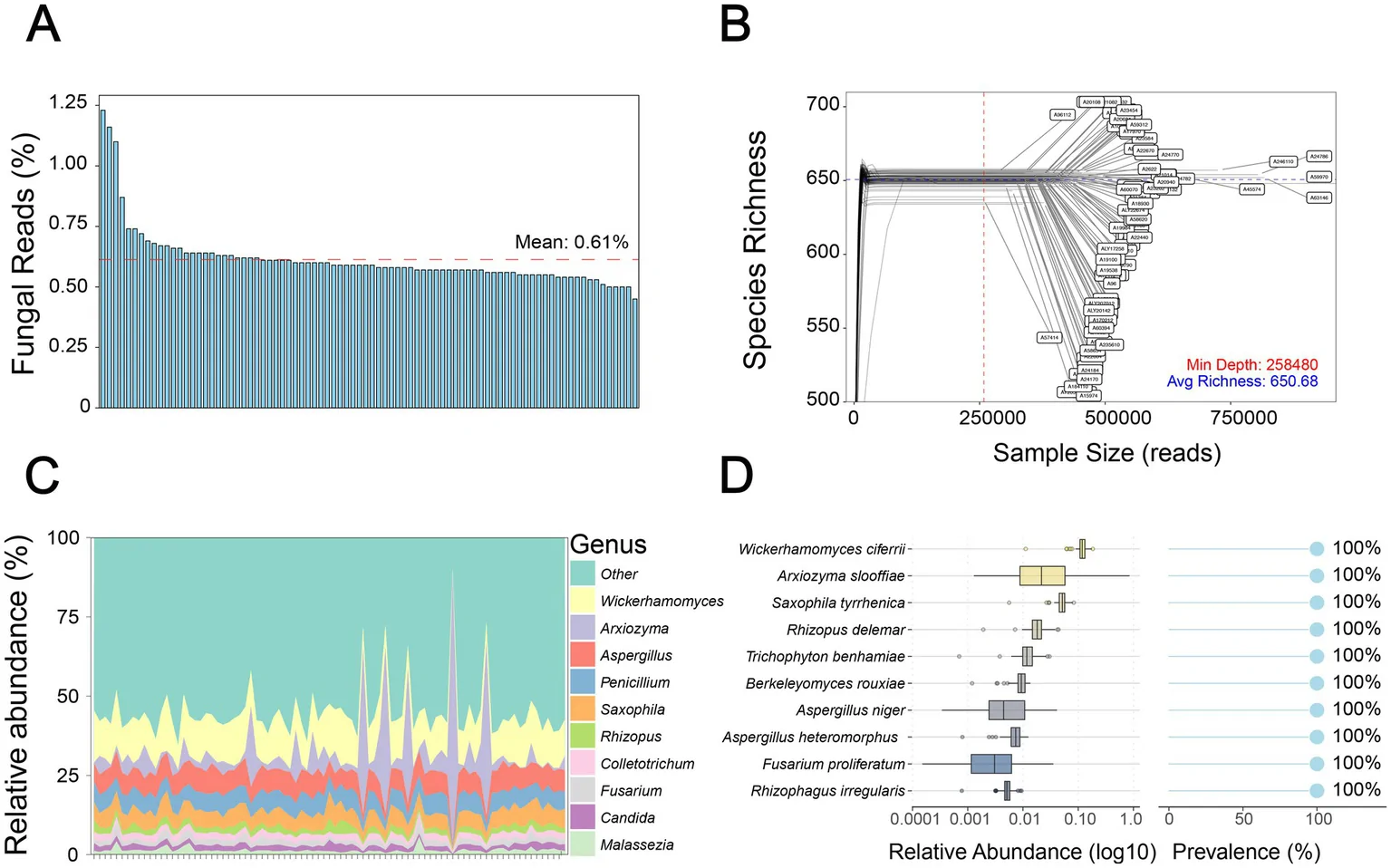
Statistical overview of gut fungal sequencing data and community profiles. **(A)** Percentage of fungal reads across samples. Blue bars represent the percentage of fungal reads per sample, with the red dashed line indicating the average percentage. **(B)** Rarefaction curves showing fungal species richness. Vertical red and horizontal blue dashed lines denote the minimum sequencing depth and the corresponding average species richness, respectively. Plateaued curves indicate adequate sequencing coverage to capture fungal diversity. **(C)** Taxonomic composition at the genus level across samples. Only the top 10 genera are displayed, with “other” representing the cumulative abundance of all remaining genera. **(D)** Relative abundance and prevalence of the top 10 core fungal species.

We characterized the gut fungal community of 85 post-weaning sows using shotgun metagenomic sequencing. There was a total of 37,388,843 reads were annotated as fungal sequences, accounting for an average of 0.61% of the total microbial reads (Figure 1A; Supplementary Table S2). Rarefaction curves showed that species richness reached a plateau at a minimum depth of 258,480 reads, which indicated that the sequencing depth was sufficient to capture the fungal diversity (Figure 1B).

At the phylum level, Ascomycota was the most predominant taxon across all samples, with a mean relative abundance exceeding 75%, followed by Basidiomycota. Other phyla, including Mucoromycota, Chytridiomycota, Microsporidia, and Zoopagomycota, were also detected at lower abundances (Supplementary Figure S1A; Supplementary Table S2). At the genus level, *Wickerhamomyces* (mean ± SD: 0.1199 ± 0.0266) and *Arxiozyma* (mean ± SD: 0.0692 ± 0.1394) were identified as the dominant genera. In addition, *Aspergillus*, *Penicillium*, *Saxophila*, *Rhizopus*, *Colletotrichum*, *Fusarium*, *Candida*, and *Malassezia* showed relatively stable distributions across samples, representing the core component of the gut fungal community in weaned sows (Figure 1C; Supplementary Table S2). Species-level analysis showed that *Wickerhamomyces ciferrii* (mean ± SD: 0.1194 ± 0.0265) was the most abundant species, while *Arxiozyma slooffiae* (mean ± SD: 0.0685 ± 0.1390) exhibited inter-individual variation in relative abundance. Additional highly abundant species included *Saxophila tyrrhenica*, *Rhizopus delemar*, *Aspergillus niger*, and *Aspergillus heteromorphus*, were consistently detected across all samples (Figure 1D; Supplementary Table S2).

### 3.2 Identification of fungal species associated with post-weaning estrus in sows

**Figure 2:**
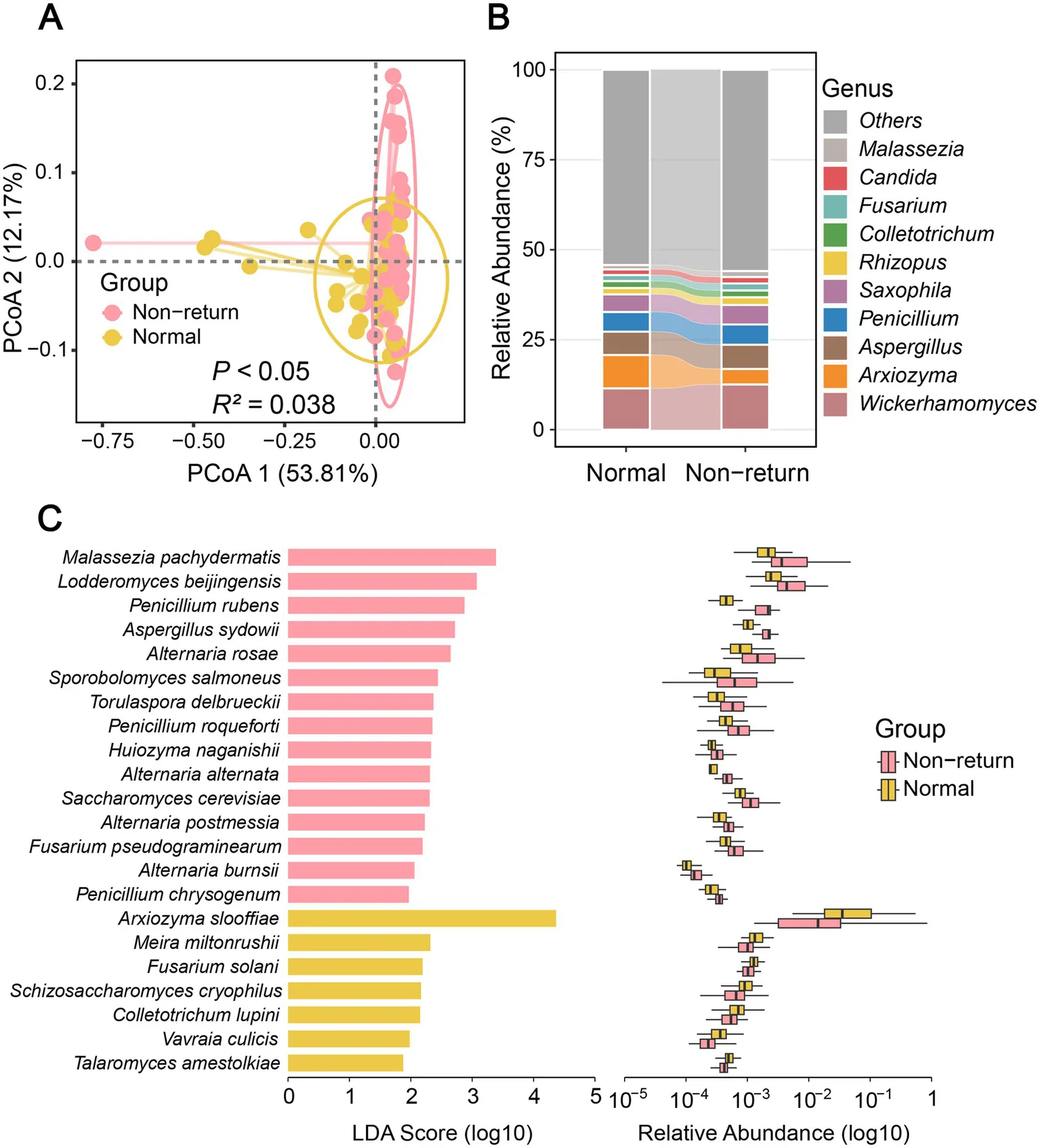
Gut mycobiome diversity and compositional differences between normal and non-return groups. **(A)** PCoA of fungal beta diversity based on Bray–Curtis dissimilarity. Percentages on axes indicate the proportion of total variance explained, and ellipses represent 95% confidence intervals. Statistical significance of group differences was determined by PERMANOVA. **(B)** Taxonomic composition at the genus level across normal and non-return groups. The top 10 most abundant genera are presented, with alluvial flows illustrating shifts in relative abundance between the two groups. **(C)** LEfSe identified differentially abundant fungal species between the two groups. (Left) Bar plots show the LDA scores of species enriched in each group (LDA ≥ 2.0, FDR < 0.05). (Right) Box plots show the relative abundances of the corresponding differential species.

To explore the association between post-weaning return to estrus and the gut fungal community in sows, the fungal diversity and composition was compared. The Shannon and Simpson metrics for alpha diversity had no significant differences between the normal and non-return groups (*p* > 0.05; Supplementary Figures S2A,B). However, beta diversity based on Bray-Curtis dissimilarities revealed significant changes in fungal community compositions between the two groups (PERMANOVA, *p* < 0.05, *R*^2^ = 0.038; Figure 2A). To rigorously evaluate whether parity acted as a confounder, an additional PERMANOVA was performed including group and parity as explanatory variables. The analysis confirmed that group remained significantly associated with fungal community composition (*R*^2^ = 0.036, *p* < 0.05), whereas parity had no significant influence (*R*^2^ = 0.016, *p* > 0.05, Supplementary Table S4).

At the genus level, we compared the relative abundances of the 10 most abundant fungal taxa between the two sow groups and observed distinct abundance patterns among several core genera. *Arxiozyma* showed a significantly higher abundance in the normal group, with a mean relative abundance of 9.26% compared to 4.29% in the non-return group (*p* < 0.05). In contrast, *Wickerhamomyces* was more abundant in the non-return group, with mean relative abundances of 12.57% versus 11.47%. Similarly, *Saxophila* and *Malassezia* exhibited significantly higher relative abundances in this group (*p* < 0.05). Other core genera showed relatively stable abundance between the two groups (Figure 2B; Supplementary Table S5).

To further identify gut fungal species associated with post-weaning return to estrus, we applied LEfSe, which revealed 22 species with significant differences between the normal and non-return groups (LDA score ≥ 2, FDR < 0.05; Figure 2C; Supplementary Table S6). In the normal group, seven fungal species were enriched, including *Arxiozyma slooffiae*, *Meira miltonrushii*, *Fusarium solani*, *and Schizosaccharomyces cryophilus*, with *Arxiozyma slooffiae* showing the highest LDA score and higher relative abundance (LDA = 4.368, FDR < 0.05; Figure 2C; Supplementary Figure S2C). In contrast, 15 fungal species were enriched in the non-return group, including *Malassezia pachydermatis, Lodderomyces beijingensis*, *Penicillium rubens*, and *multiple Alternaria species* (*Alternaria rosae*, *Alternaria alternata*, *Alternaria postmessia,* and *Alternaria burnsii*). Importantly, the associations of these 22 differential taxa with estrus return were further validated using multivariate logistic regression models adjusting for parity. The results confirmed that all 22 taxa exhibited enrichment directions consistent with the LEfSe findings, and the vast majority remained statistically significant after adjustment (Supplementary Table S7).

### 3.3 Cross-kingdom fungal-bacterial interaction associated with post-weaning return to estrus

**Figure 3:**
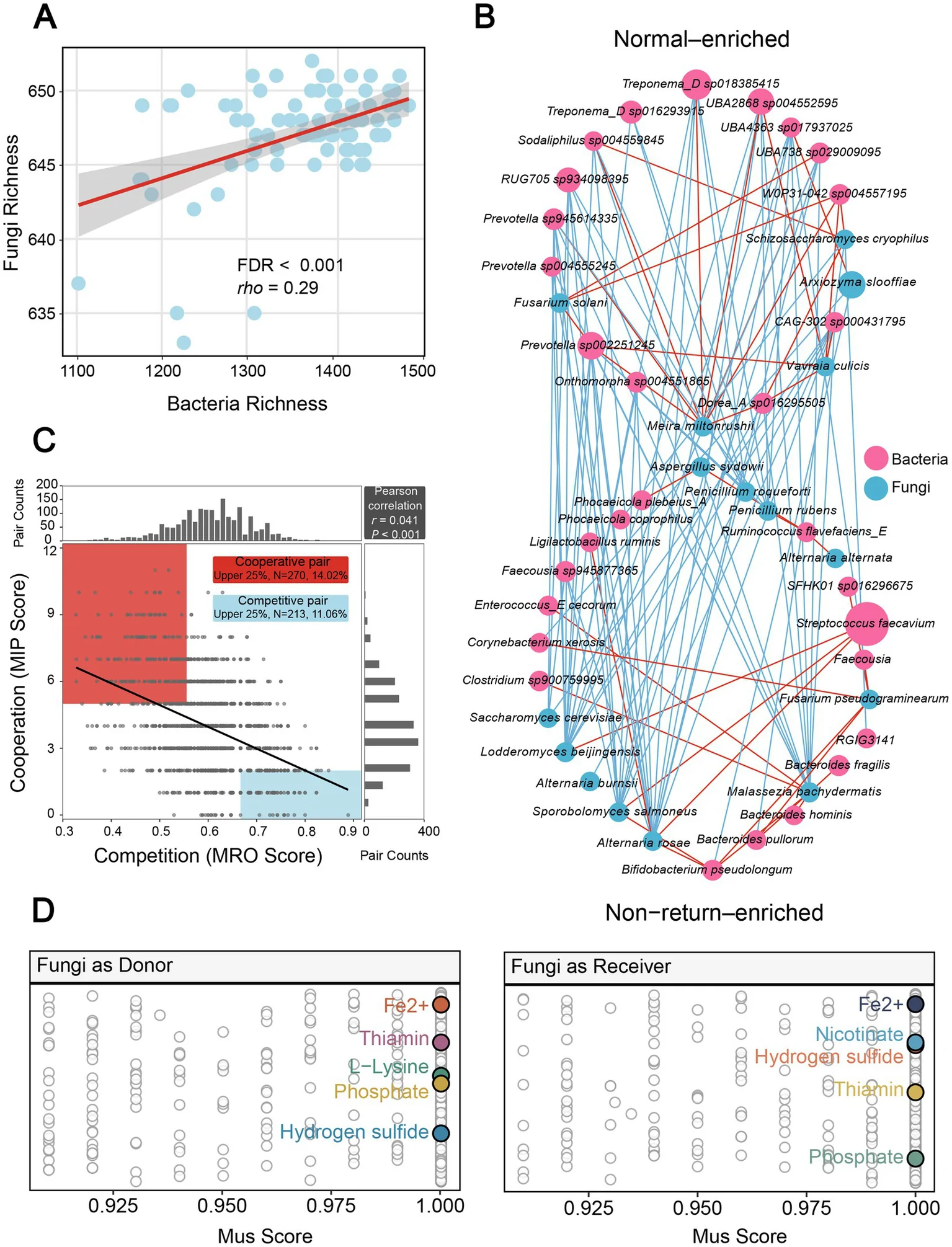
Fungal–bacterial associations and metabolic interactions associated with return to estrus. **(A)** Positive correlation between bacterial and fungal species richness. Linear regression (red line) and 95% confidence interval (shaded gray) illustrate the relationship across all samples. The correlation was assessed by Spearman correlation, and statistical significance was determined using FDR-adjusted *p*-values. **(B)** Association network of estrus-associated fungal and bacterial species. Nodes represent fungal (blue) or bacterial (pink) species, with node size reflecting average relative abundance. Blue and red edges denote positive and negative correlations, respectively. Only significant correlations with |*rho*| ≥ 0.4 and FDR < 0.05 are shown. Nodes are spatially arranged to highlight interaction patterns. **(C)** Scatter plot showing the distribution of MRO and MIP scores across all species pairs (*n* = 2,112) formed by 22 differential fungal species and 48 bacterial MAGs between the two groups. Red and blue regions indicate cooperative and competitive species pairs, respectively. Linear regression (solid line) illustrates the relationship between MRO and MIP scores, and the correlation was assessed by Pearson correlation. **(D)** Metabolic exchange potential between fungi and bacteria inferred from SMETANA. Left and right panels show fungi acting as metabolic donors and receivers, respectively. Each gray circle represents a predicted exchanged metabolite with MUS scores > 0.9. The top five metabolites with the highest occurrence frequency across all fungal-bacterial species pairs are highlighted.

The gut microbiota is a complex ecosystem where fungal and bacterial communities engage in cross-kingdom interactions that are crucial for host health (Wu et al., 2021; Liu et al., 2025). We first explored the cross-kingdom associations between the gut fungal and bacterial communities in post-weaning sows. A significant positive correlation was observed between fungal and bacterial species richness (Spearman’s *rho* = 0.29, FDR < 0.05), suggesting a mutualistic relationship between the two kingdoms in the post-weaning sow gut (Figure 3A). To further characterize the cross-kingdom interactions associated with post-weaning return to estrus, we focused on fungal and bacterial taxa that were associated with return to estrus and constructed a correlation network based on 22 differential fungal species and 48 bacterial HQMAGs. Overall, fungal and bacterial taxa enriched in the same group tended to be positively correlated, whereas those enriched in different groups exhibited negative correlations. For example, fungal species enriched in the normal group, *Arxiozyma slooffiae*, *Schizosaccharomyces cryophilus, Vavraia culicis*, and *Meira miltonrushii* were positively correlated with bacterial taxa including *Prevotella* spp., *Treponema* spp., and *Dorea* sp., forming a positively correlated module that accounted for 48.8% of all positive correlations. Likewise, fungi enriched in the non-return group, such as *Alternaria* spp., *Penicillium* spp., *Malassezia pachydermatis*, and *Fusarium pseudograminearum* showed positive correlations with bacterial taxa including *Bacteroides fragilis* and *Enterococcus_E cecorum*, forming a module that represented 51.2% of the total positive correlations. In contrast, taxa enriched in the normal group were consistently negatively correlated with those enriched in the non-return group, accounting for all observed negative correlations. For instance, *Arxiozyma slooffiae* showed negative associations with *Corynebacterium xerosis* and *Bifidobacterium pseudolongum* (Figure 3B; Supplementary Table S8).

To further understand the associations reflected in the cross-kingdom network, the microbial associations were examined from the perspective of metabolic interactions. Using SMETANA, we assessed the metabolic interactions between these bacterial and fungal taxa by quantifying MIP and MRO, which reflect the potential for metabolic cooperation and resource competition, respectively. Based on the distribution patterns of MIP and MRO, we identified 213 competitive species pairs (11.06%, MIP ≤ 2 and MRO ≥ 0.667) and 270 cooperative pairs (14.02%, MIP ≥ 5 and MRO ≤ 0.556), with MIP negatively correlated with MRO (Pearson’s *r* = −0.421*, p* < 0.001) (Figure 3C). Notably, 63 of the 483 species pairs identified by SMETANA were also present in the correlation network. Among these, 10 pairs (15.9%) showed concordant directions, whereas 53 pairs (84.1%) exhibited opposite trends (Supplementary Figure S3A), suggesting that correlations do not necessarily reflect the underlying metabolic interactions. We further examined the metabolites potentially exchanged between these fungal and bacterial taxa and found that Fe^2+^, thiamine, L-lysine, phosphate, hydrogen sulfide, and nicotinate were commonly exchanged (Figure 3D).

### 3.4 Combined fungal and bacterial biomarkers enhance discriminative performance for post-weaning estrus return

**Figure 4:**
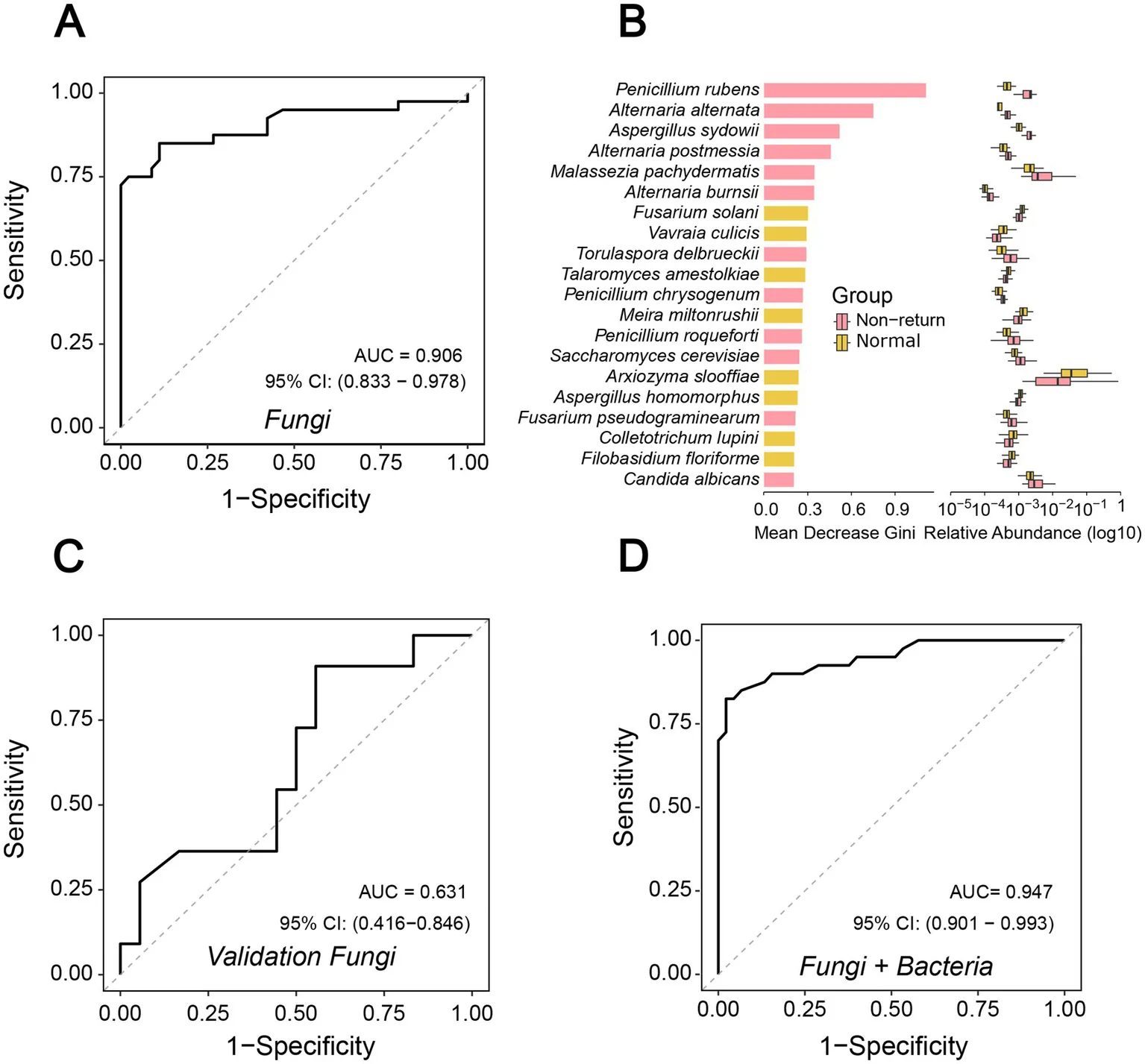
Construction and validation of random forest models for distinguishing normal and non-return groups. **(A)** ROC curve showing the classification performance of the fungal-based model using leave-one-out cross-validation in the discovery cohort (*n* = 85). **(B)** Identification of key fungal species contributing to model classification performance in the discovery cohort. (Left) Bar plots show the top 20 fungal species ranked by mean decrease in Gini (MDG) index. (Right) Box plots show the relative abundances of these species. **(C)** ROC curve of the fungal-based diagnostic model in the independent validation cohort (*n* = 29). **(D)** ROC curve of the combined model integrating fungal taxa and high-quality bacterial MAGs in the discovery cohort using leave-one-out cross-validation, showing improved discriminative performance over the fungal-only model.

To evaluate the potential of the gut fungi as biomarkers for distinguishing return to estrus in post-weaning sows, a random forest classification model was constructed based on fungal species relative abundances. Receiver operating characteristic (ROC) analysis showed that the model achieved a high discriminative performance, with an area under the curve (AUC) of 0.906 (95% CI: 0.833–0.978) and an accuracy of 0.871 (Figure 4A; Supplementary Table S9). Feature importance analysis identified the top 20 fungal species contributing to the model performance. Notably, several of these species, including *Penicillium rubens*, *Alternaria alternata*, *Arxiozyma slooffiae*, and *Vavraia culicis*, were also identified as differential fungi by LEfSe analysis, further supporting their potential as biomarkers of post-weaning estrus return in sows (Figure 4B). To evaluate the generalizability of the fungal model, the finalized RF model trained on the entire discovery cohort was tested on an independent validation cohort. The model showed moderate performance in the validation cohort (AUC = 0.631, 95% CI: 0.416–0.846; accuracy = 0.552; Figure 4C; Supplementary Table S9).

Growing evidence suggests that integrating cross-kingdom microbial features can improve the classification performance (Liu et al., 2022; Huang et al., 2023). To test this, we incorporated 48 differential bacterial HQMAGs into the model. In the discovery cohort, the combined panel of fungal and bacterial features exhibited enhanced performance (AUC = 0.947, 95% CI: 0.901–0.993; accuracy = 0.894), compared with the fungal panel alone (Figure 4D; Supplementary Table S9). Crucially, we also evaluated the performance of the combined model in the independent validation cohort. The multi-kingdom panel yielded an AUC of 0.657 (95% CI: 0.454–0.860), which was slightly higher than that of the fungal-only model (AUC = 0.631), although other classification metrics (e.g., sensitivity, specificity, PPV, and NPV) remained comparable or were slightly lower (Supplementary Figure S5; Supplementary Table S9). Overall, while the fungal-based model demonstrated strong discriminatory performance in the discovery cohort, its generalizability in the independent validation cohort was relatively limited. Nonetheless, the incorporation of bacterial features consistently improved the overall AUC across both cohorts.

### 3.5 Alterations in the gut fungi correlate with metabolic and functional changes associated with post-weaning estrus return

**Figure 5:**
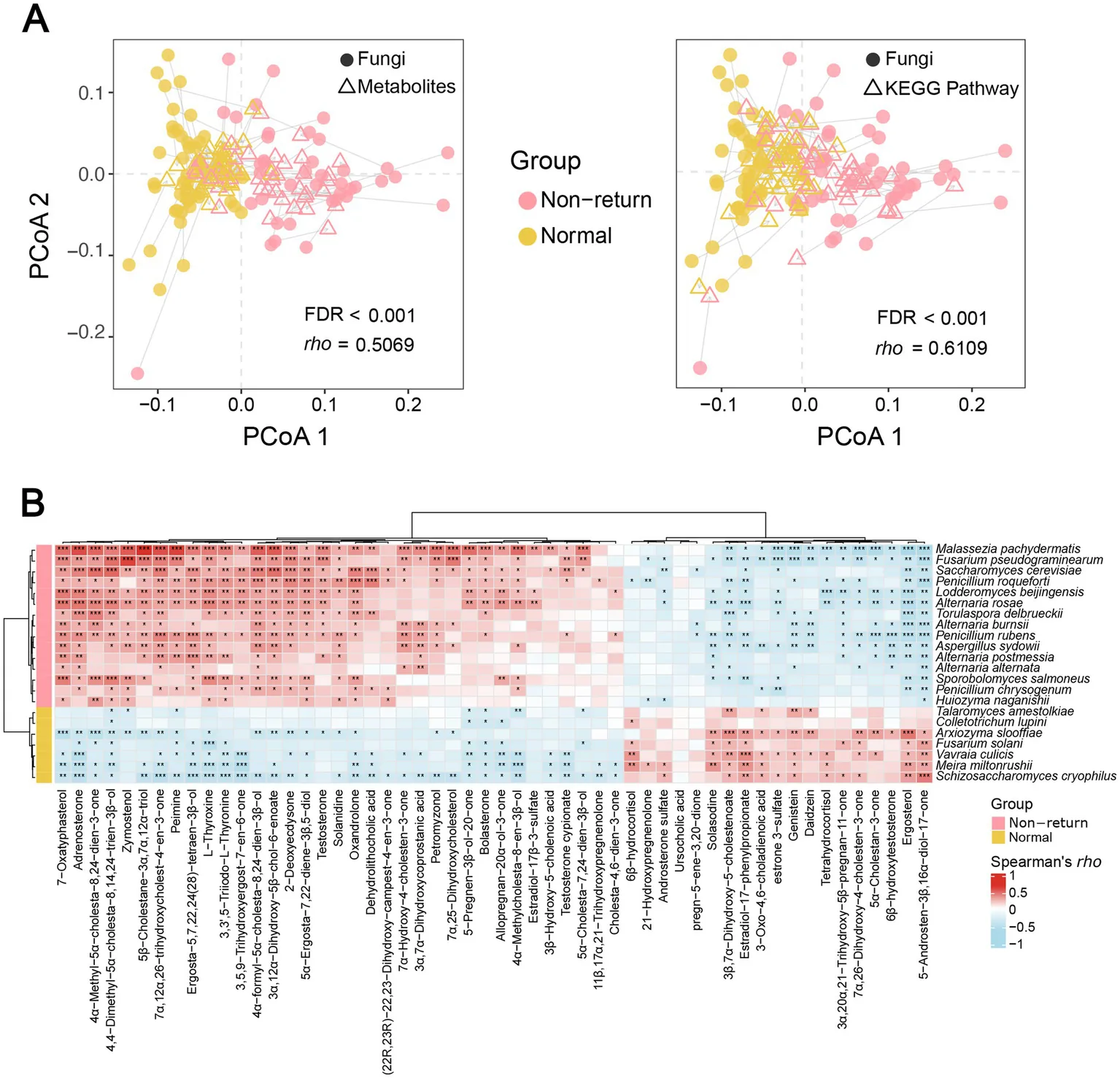
Associations between gut fungal taxa, metabolites, and functional pathways. **(A)** Procrustes analysis showing the concordance between fungal community structure and fecal metabolite profiles (left) or KEGG pathway abundances (right). Dots represent fungal community ordination, triangles represent metabolite or pathway ordination, and lines connect the same samples across the two ordination spaces, with shorter residuals indicating higher configurational similarity between the two data layers. Statistical significance was evaluated using the Protest permutation test (9,999 permutations). **(B)** Heatmap showing the correlations between differential fungal species and hormones and hormone-related compounds. Fungal species (rows) are colored by enrichment group (yellow, normal; pink, non-return). The color gradient represents correlation coefficients, with red indicating positive correlations and blue indicating negative correlations. Correlations were assessed by Spearman correlation. Statistical significance is denoted by asterisks: *p < 0.05, **p < 0.01, ***p < 0.001.

Changes in the gut mycobiome composition are closely associated with shifts in host physiological state (Iliev and Leonardi, 2017; Zhao et al., 2023). To explore whether gut fungi associated with return to estrus are linked to specific microbial functional pathways and metabolic profiles, we evaluated the correspondence between 22 differential fungal species identified in the current study and both the 54 fecal hormones and hormone-related compounds and the 60 KEGG pathways previously identified in our bacterial study (Liu et al., 2023) using Procrustes analysis. The results revealed significant concordance between the gut fungal community and both fecal hormones and hormone-related compounds (*rho* = 0.5069, FDR < 0.001) and microbial functional pathways (*rho* = 0.6109, FDR < 0.001) (Figure 5A).

To further delineate the specific associations underlying this concordance, Spearman correlation analysis was performed and showed that fungal species were significantly positively correlated with hormones and hormone-related compounds, and functional pathways enriched in the same group. In the normal group, for example, *Arxiozyma slooffiae*, *Meira miltonrushii*, *Vavraia culicis*, and *Fusarium solani* showed strong positive associations with phytoestrogens, including daidzein and genistein, as well as estrone 3-sulfate and estradiol-17-phenylpropionate. Consistently, fungal species enriched in the normal return group were positively correlated with reproductive hormone–related pathways, including steroid hormone biosynthesis, ovarian steroidogenesis, and estrogen signaling pathway. In contrast, in the non-return group, fungal species such as *Malassezia pachydermatis*, *Alternaria rosae*, and *Saccharomyces cerevisiae* were significantly positively correlated with adrenosterone, testosterone, bolasterone, 5-pregnen-3β-ol-20-one, and allopregnan-20α-ol-3-one. Moreover, these fungal species were positively associated with steroid degradation pathways enriched in this group but negatively associated with the reproductive hormone-related pathways enriched in the normal group (Figure 5B; Supplementary Figure S4A; Supplementary Tables S10, S11).

## 4 Discussion

Research into post-weaning estrus return in sows has predominantly focused on the gut bacterial community. Our previous findings demonstrated that specific bacterial taxa influence return to estrus by regulating the metabolism of sex steroid hormones (Liu et al., 2023). Complementing these bacterial-centric insights, the present study provides the first systematic characterization of the gut fungal community in post-weaning sows and establishes a link between fungal community composition and return to estrus. We identified specific differentially abundant fungal species between the two groups and demonstrated significant cross-kingdom interactions between fungal and bacterial taxa associated with estrus return. Furthermore, multi-omics integration revealed extensive correlations between the gut fungi, hormones and hormone-related compounds, and microbial functional pathways. Although the underlying causal mechanisms remain to be elucidated, these findings collectively demonstrate that the gut mycobiome and its cross-kingdom interactions are significantly associated with post-weaning estrus return, highlighting the previously overlooked yet pivotal importance of the gut mycobiome in sow reproductive performance.

Consistent with previous reports that fungi represent a small minority of the mammalian gut microbiome (Nash et al., 2017; Huang et al., 2024), the average fungal read fraction observed in this study was 0.61%. Notably, this proportion is significantly higher than the 0.036% average reported in a recent large-scale survey of 750 porcine samples covering diverse ages, genders, breeds, and health statuses (Wei et al., 2026). This enhanced capture rate is primarily attributed to our superior sequencing depth (20–22 Gb per sample), which is approximately double the typical depth (10–12 Gb) used in many large-scale datasets, providing greater sensitivity for characterizing the low-abundance gut mycobiome. In terms of community composition, the predominance of Ascomycota and Basidiomycota in our study is highly consistent with the findings of that large-scale survey, where these two phyla, along with Mucoromycota, represented over 99.0% of the mapped fungal sequences (Wei et al., 2026). Similar dominance has also been observed in healthy and diarrheal Tibetan piglets (Kong et al., 2021), as well as across various breeds such as Chenghua and Yorkshire (Li et al., 2020). Our results further reveal that specific fungal taxa are associated with estrus return in post-weaning sows. At the genus level, the normal group was characterized by the predominance of *Arxiozyma*, with *Arxiozyma slooffiae* being the most abundant species. *Kazachstania* (currently *Arxiozyma*) species are widely recognized as core commensals of the healthy pig gut mycobiota, potentially contributing to intestinal homeostasis (Urubschurov et al., 2017; Ramayo-Caldas et al., 2020; Hu et al., 2023). Specifically, *Kazachstania slooffiae* has been reported to promote intestinal epithelial glycolysis (Hu et al., 2023), which may facilitate energy harvest and potentially support estrus return in post-weaning sows. Conversely, the non-return group exhibited significant enrichment of several potentially pathogenic fungal taxa, including *Alternaria* spp., *Penicillium* spp. and *Malassezia pachydermatis*. These genera are frequently associated with fungal dysbiosis and have been linked to exacerbated intestinal inflammation (Wheeler et al., 2016; Li et al., 2018; Aykut et al., 2019). Furthermore, *Fusarium pseudograminearum*, a recognized pathogenic and mycotoxin-producing fungus whose spores have been reported to perturb host gut microbial and metabolic homeostasis (Wang et al., 2024), was also enriched in the non-return group. These findings suggest that specific fungal species are associated with post-weaning estrus return in sows. However, whether these taxa directly influence post-weaning return to estrus in sows remains to be confirmed through further functional studies.

The gut microbiota is a complex ecosystem in which microorganisms collectively support intestinal homeostasis through metabolic crosstalk, ecological interactions, and immune modulation (Xie et al., 2023; Wang et al., 2025; Zuo, 2025). Our network analysis revealed that fungal and bacterial taxa enriched in the same group tended to be positively correlated, whereas those enriched in different groups exhibited negative associations. These findings indicate that the observed cross-kingdom interaction patterns are significantly associated with post-weaning estrus return in sows. To investigate the potential metabolic interactions underlying these associations, genome-scale metabolic modeling was employed. Specifically, we identified Fe^2+^, thiamine, L-lysine, phosphate, H_2_S, and nicotinate as the most frequently exchanged metabolites between estrus-associated fungal and bacterial taxa. Thiamine and nicotinate serve as essential coenzymes in redox reactions and energy metabolism (Yang and Sauve, 2016; Folda et al., 2025). The exchange of these vitamins B allows microorganisms to establish mutualistic relationships (Cooper et al., 2019). Furthermore, H_2_S act as crucial cross-species mediators that influences bacterial respiration, enzymatic activity, and redox homeostasis, thereby influencing microbial community structure and function (Tran et al., 2023; Tikhomirova et al., 2024; Nonoyama et al., 2026). Due to the extreme scarcity of free iron in the anaerobic gut environment, fungi and bacteria mainly rely on reductive iron uptake and siderophore-mediated iron uptake (Kosman, 2003; Carver, 2018). In this process, microbial taxa may compete for limited iron and exploit siderophores produced by others, generating metabolic dependencies and potential mutualism (Folkesson et al., 2012; Speirs et al., 2012). These mechanisms highlight the central role of metabolic exchange, particularly involving iron and vitamins, in cross-kingdom interactions among fungal and bacterial taxa associated with estrus return. However, it is noteworthy that while our analysis revealed that cooperative metabolic interactions generally predominated over competitive ones, aligning with the framework that metabolic dependency promotes species coexistence through complementary metabolic functions (Zelezniak et al., 2015). Notably, only a subset of the metabolically predicted interacting pairs was consistent with the abundance-based correlation network. This discrepancy aligns with previous reports that abundance-based correlation analyses provide limited evidence for true interactions and may be confounded by shared environmental responses or niche overlap (Blanchet et al., 2020; Chaffron et al., 2021). Furthermore, such inconsistencies likely arise because genome-scale metabolic models infer the potential for metabolite exchange based on genomic capabilities, whereas abundance-based correlations reflect realized ecological outcomes under specific environmental conditions. For example, while metabolite cross-feeding allows microorganisms to utilize resources released by other community members (Ponomarova and Patil, 2015; Goldford et al., 2018), these interactions do not always translate into detectable shifts in microbial abundance. Moreover, the balance between cooperation and competition is highly dynamic; under resource-limited conditions, competitive interactions may outweigh the benefits of metabolite exchange (Coyte et al., 2015), potentially masking predicted cooperative relationships in abundance-based correlation analyses. In summary, our findings underscore that statistical associations do not necessarily reflect underlying metabolic interactions, highlighting the need for more refined metabolic modeling to better capture the interaction mechanisms between cross-kingdom microbial taxa. Nevertheless, these results are inferred from correlation patterns and metabolic modeling, and further experimental validation is needed to confirm their causal and functional relevance.

In the normal group, specific fungal taxa, such as *Arxiozyma slooffiae* and *Vavraia culicis*, were positively associated with phytoestrogens (e.g., daidzein and genistein). Phytoestrogens are well-known to exert weak estrogenic activity and can influence host physiology via estrogen receptors (Mueller et al., 2004). While their gut biotransformation has been primarily attributed to bacterial activity (Hameed et al., 2020), the observed correlations suggest that the gut mycobiome may be associated with the phytoestrogen-related metabolic processes. Meanwhile, these fungal taxa also exhibited positive associations with functional pathways such as sex steroid hormone biosynthesis, ovarian steroidogenesis, and estrogen signaling, all of which are critical for the regulation of estrus return (Madej et al., 2005; Liu et al., 2023). Conversely, the non-return group was characterized by the enrichment of fungal species such as *Malassezia pachydermatis* and *Alternaria rosae*, which exhibited positive correlations with androgens (e.g., testosterone and adrenosterone) and progestogen-related compounds (e.g., allopregnan-20α-ol-3-one and 5-pregnen-3β-ol-20-one). Considering that androgen accumulation can antagonize estrogen to interfere with follicle development (Hammes and Levin, 2019), and elevated progestogens may exert negative feedback on the HPO axis to inhibit FSH secretion (Skinner et al., 1998), these associations suggest a potential relationship between fungal taxa and the reproductive hormone homeostasis of post-weaning sows. Furthermore, the positive correlation of fungal taxa with steroid degradation pathways, alongside their negative associations with reproductive hormone-related pathways enriched in the normal group, implies that this fungal profile may be unfavorable for post-weaning estrus return. Taken together, these findings revealed extensive associations between the gut fungi and hormones and hormone-related compounds and functional pathways, suggesting that the gut mycobiota are associated with reproductive hormone homeostasis and return to estrus in post-weaning sows.

Several limitations of this study should be acknowledged. First, while our study has established a relationship between the gut mycobiome and post-weaning estrus return, these findings remain correlational and do not demonstrate a causal role for the gut mycobiome in regulating estrus return. The lack of longitudinal reproductive hormone measurements (e.g., estrogen and progesterone) further limits our ability to directly bridge gut fungal alterations with host endocrine dynamics. Additionally, the relatively small sample size of the independent validation cohort (*n* = 29) and potential farm-level variations (e.g., subtle differences in management or environmental microbial exposure) may have constrained the statistical power to fully capture the diagnostic potential of certain fungal biomarkers, potentially contributing to the moderate generalizability observed in our models. Furthermore, we employed an extreme-phenotype design by excluding a small proportion of sows with intermediate WEI (8–14 days) to maximize the signal-to-noise ratio for identifying core microbial signatures associated with estrus return. Future studies incorporating multi-farm cohorts across the full range of WEI will be essential to better disentangle these environmental effects from true biological signals and to determine whether sows with intermediate phenotypes harbor transitional microbial signatures. Beyond these design constraints, many gut-resident fungi remain uncultured or lack high-quality reference genomes, which may lead to an underestimation of fungal diversity. By using a curated RefSeq-based database, some low-abundance or uncharacterized fungal species may not have been captured, reflecting a broader challenge in the field. Future research into the swine gut mycobiome will necessitate enhanced culturomics and the development of high-quality reference genomes to bridge these gaps. Finally, whether these associations reflect direct fungal action or are mediated through complex cross-kingdom interactions with bacteria warrants further experimental validation. Despite these limitations, our results highlight the association between the gut mycobiome and sow reproductive performance, providing a foundation for future functional studies to clarify the underlying mechanisms.

## 5 Conclusion

In conclusion, this study provides the first comprehensive gut mycobiome profiling in post-weaning sows and reveals significant associations with return to estrus. We identified differentially abundant fungal species between the two groups and demonstrated fungal and bacterial cross-kingdom interactions associated with return to estrus. Multi-omics integration revealed extensive associations between gut fungi and reproductive hormone-related compounds and functional pathways. Although the causality and underlying mechanisms need to be further confirmed, this study provides valuable evidence that gut mycobiota may be an important component of the gut microbiome associated with sow post-weaning reproductive performance.

## Data Availability

The metagenomic sequencing data generated in this study have been submitted to the GSA database under accession numbers: CRA008698 and are available at: https://download.cncb.ac.cn/gsa2/CRA008698.

## Glossary

WEI: Weaning-to-estrus interval
NPD: Non-productive days
PSY: Piglets per sow per year
ITS: Internal transcribed spacer
BH: Benjamini-hochberg
FDR: False discovery rate
MAGs: Metagenome-assembled genomes
GEMs: Genome-scale metabolic models
MIP: Metabolic interaction potential
MRO: Metabolic resource overlap
MUS: Metabolite uptake scores
LEfSe: Linear discriminant analysis effect size
LDA: Linear discriminant analysis
AUC: Area under the ROC curve
ROC: Receiver operating characteristic
LOOCV: Leave-one-out cross-validation
KEGG: Kyoto Encyclopedia of Genes and Genomes
PcoA: Principal coordinates analysis
PPV: Positive predictive value
NPV: Negative predictive value
