# Supplementary material for: Associations of the gut mycobiome and its cross-kingdom interactions with estrus return in post-weaning sows revealed by metagenomic analysis
Source: Front Microbiol. 2026 Sep 3;17:1892244. doi: 10.3389/fmicb.2026.1892244 (PMC13582324; doi:10.3389/fmicb.2026.1892244)
Supplement: Supplementary file 1 [file Supplementary_file_1.DOCX]

**Associations of the gut mycobiome and its cross-kingdom interactions with estrus return in post-weaning sows revealed by metagenomic analysis**

Pan Jiang, Mengqing Zhou, Yuhong Liao, Wenbo Du, Min Liu*

*National Key Laboratory of Swine Genetic Improvement and Germplasm Innovation, Jiangxi Agricultural University, Nanchang 330045, China*

Pan Jiang: [jiangpan204@gmail.com](mailto:jiangpan204@gmail.com)

Mengqing Zhou: [MengqingZhou1996@hotmail.com](mailto:MengqingZhou1996@hotmail.com)

Yuhong Liao: [18079012735@163.com](mailto:18079012735@163.com)

Wenbo Du: [Duwb4125@gmail.com](mailto:Duwb4125@gmail.com)

Min Liu: [liumin6751@163.com](mailto:liumin6751@163.com)

***Corresponding authors:** Min Liu

National Key Laboratory of Swine Genetic Improvement and Germplasm Innovation, Jiangxi Agricultural University, Nanchang 330045, China

Email: [liumin6751@163.com](mailto:liumin6751@163.com)

Tel: 0086-791-83813080; Fax: 0086-791-8390018

**Figure S1.** Taxonomic composition at the phyla (A) and species (B) levels across samples. Only the top 10 species are displayed, with "Other" representing the cumulative abundance of all remaining species.

**Figure S2.** **(****A, B)** Box plots of alpha diversity indices: (A) Shannon index and (B) Simpson index. No significant differences were observed between the two groups. **(C)** Differences in the relative abundance of *Arxiozyma slooffiae* between the two groups. **(D)** Heatmap of LEfSe-identified differential fungal species. Samples are ordered by group. Species are hierarchically clustered (left dendrogram), yielding two major blocks consistent with group separation. Abundances were log10-transformed and robust Z-score normalized per species (centered by median, scaled by MAD × 1.4826; IQR used when MAD = 0). Colors (blue to red) indicate low to high relative abundance.

**Figure S3. (A)** Comparison between correlation networks and GEMs for 22 fungal species and 48 MAGs. (Left) Bar plot showing the number of species pairs identified by correlation analysis (Correlation network, defined by |*rho*| ≥ 0.4 and FDR < 0.05) and those predicted by GEMs (GEMs, including competitive and cooperative pairs defined in this study.). (Right) Pie plot showing the number and percentage of Correlation species pairs that align with GEMs. “Direction consistent with GEMs” indicates that the correlation direction fully agrees with the interaction type predicted by GEMs (i.e., positive correlation corresponds to cooperation, and negative correlation corresponds to competition). “Direction inconsistent with GEMs” indicates that the pairs is present in the GEMs predictions but shows the opposite direction. “Not detected in GEMs” indicates that the correlation-identified pair could not be mapped to any GEM-predicted pairs.

**Figure S4. (A)** Heatmap showing the correlations between differential fungal species and KEGG pathway. Fungal species (rows) are colored by enrichment group (yellow, normal; pink, non-return). The color gradient represents correlation coefficients, with red indicating positive correlations and blue indicating negative correlations. Correlations were assessed by Spearman correlation. Statistical significance is denoted by asterisks: **P* < 0.05, ***P* < 0.01, ****P* < 0.001.

**Figure S5.** ROC curve of the combined model integrating fungal taxa and high-quality bacterial MAGs in the independent validation cohort.
